# Superinfection exclusion and the long-term survival of honey bees in Varroa-infested colonies

**DOI:** 10.1038/ismej.2015.186

**Published:** 2015-10-27

**Authors:** Gideon J Mordecai, Laura E Brettell, Stephen J Martin, David Dixon, Ian M Jones, Declan C Schroeder

**Affiliations:** 1 Viral Ecology, Marine Biological Association, Plymouth, UK; 2 School of Biological Sciences, University of Reading, Reading, UK; 3 School of Environment and Life Sciences, The University of Salford, Manchester, UK

## Abstract

Over the past 50 years, many millions of European honey bee (*Apis mellifera*) colonies have died as the ectoparasitic mite, *Varroa destructor*, has spread around the world. Subsequent studies have indicated that the mite's association with a group of RNA viral pathogens (Deformed Wing Virus, DWV) correlates with colony death. Here, we propose a phenomenon known as superinfection exclusion that provides an explanation of how certain *A. mellifera* populations have survived, despite *Varroa* infestation and high DWV loads. Next-generation sequencing has shown that a non-lethal DWV variant ‘type B' has become established in these colonies and that the lethal ‘type A' DWV variant fails to persist in the bee population. We propose that this novel stable host-pathogen relationship prevents the accumulation of lethal variants, suggesting that this interaction could be exploited for the development of an effective treatment that minimises colony losses in the future.

## Introduction

The recent global decline of the European honey bee (*Apis mellifera*) populations (Ratnieks and Carreck, 2010; Schroeder and Martin, 2012) is of grave concern because of their role as pollinators which contribute an estimated $225 billion to the global economy (Gallai *et al.*, 2009). For over half a century, the global spread of the ectoparasitic mite, *Varroa destructor*, has resulted in the death of many millions of managed and feral honey bee colonies (Martin *et al.*, 2012; Schroeder and Martin, 2012; Thompson *et al.*, 2014). The mite has introduced a new viral transmission route that has dramatically altered the viral landscape (Martin *et al.*, 2012). This has resulted in a massive loss of diversity in Deformed Wing Virus (DWV) (Martin *et al.*, 2012), the pathogen now linked with the collapse of honey bee colonies (Highfield *et al.*, 2009; Di Prisco *et al.*, 2011). However, prior to *Varroa* spread, DWV stably co-existed with honey bees (Martin *et al.*, 2012) albeit at viral loads many orders of magnitude lower than is now observed (Martin *et al.*, 2012; Mondet *et al.*, 2014). For example, the recent arrival of *Varroa* into the Hawaiian honey bee population was accompanied by a million fold increase in the viral load of DWV, loss of DWV diversity and the predominance of a single highly virulent DWV variant (type A) (Martin *et al.*, 2012). These landscape scale changes have also been demonstrated at the individual honey bee level within the UK honey bee population. For example, Ryabov *et al.* (2014) demonstrated the dominance of a single variant of DWV when a mixture of viral strains were injected into developing pupae leading to a rapid loss of DWV diversity and million fold increase in viral loads.

DWV is a rapidly evolving group of closely related variants (de Miranda and Genersch, 2010), commonly referred to as a quasispecies (Domingo and Holland, 1997; Lauring and Andino, 2010). Within the DWV quasispecies, there are several master variants, each with its own swarm of variants. Each variant can form potential recombinants with other variants, within a swarm and between master variants. Kakugo virus is a variant of the DWV type A that differs from the master sequence (Lanzi *et al.*, 2006) by 6% in the non-structural coding region (Fujiyuki *et al.*, 2006; Baker and Schroeder, 2008), whereas Varroa destructor Virus-1 (VDV-1) (Ongus *et al.*, 2004) is genetically dissimilar to DWV type A (84% genome identity) and is referred to as DWV type B (Martin *et al.*, 2012). Notably, both DWV type A and B master variants are able to replicate within mites and honey bees, and both have been detected in honey bees in the absence of *Varroa* (Yue and Genersch, 2005; Zioni *et al.*, 2011; Martin *et al.*, 2012). Recombinants between the variants have been reported (Moore *et al.*, 2011; Zioni *et al.*, 2011; Ryabov *et al.*, 2014) and a novel recombinant between DWV type A and a new DWV master variant, type C, has also been recently discovered (Mordecai *et al.*, 2015), suggesting that they are part of the same quasispecies and share a recent common ancestor. DWV type A has been detected in honey bee populations around the world and in the presence of *Varroa* leads to colony death (Di Prisco *et al.*, 2011; Martin *et al.*, 2012), whereas there are no known instances of type B being linked to colony death. The role of the new type C in overwintering colony losses is currently unclear (Highfield *et al.*, 2009; Mordecai *et al.*, 2015).

In the early 1990s, *Varroa* swept across the UK and was followed by widespread colony deaths 1–3 years later. To ensure the long-term survival of their honey bee colonies, beekeepers in *Varroa*-infested countries manage *Varroa* populations (Sumpter and Martin, 2004), largely through chemical methods. Nonetheless, there are reports of rare isolated untreated *A. mellifera* colonies of European origin thriving despite *Varroa* infestation, including cases on an island in Brazil (DeJong and Soares, 1997) and in small forest patches in France (Conte *et al.*, 2007) and New York, USA (Seeley, 2007). The survival of these colonies is well documented and not questioned, however, the mechanism by which tolerance to *Varroa* and its association with DWV is maintained remains elusive. In the UK, a small number of beekeepers opted not to control their mite populations and, in most cases, lost their bees. However, one UK beekeeper, Ron Hoskins, initiated a closed breeding programme from colonies that survived the initial *Varroa* infestation and this isolated population of up to 40 colonies persists in Swindon, central England, without chemical control of *Varroa* (http://www.swindonhoneybeeconservation.org.uk/). The aim of this study was to assess the viral landscape in this apiary thereby determining whether the colonies remained disease-free owing to an absence of DWV. We show here that the Swindon apiary is dominated by an avirulent DWV type B master variant with the concomitant absence of the virulent DWV type A master variant. Taken together, these data suggest that a phenomenon known as superinfection exclusion (SIE) (Salaman, 1933; Labrie *et al.*, 2010) is a plausible explanation for why this isolated UK honey bee population has survived, despite *Varroa* infestation and high DWV loads.

## Material and methods

### Sample collection

Pooled asymptomatic honey bees were collected from sites in Hawaii and from the Swindon Apiary. A time series of three hives in Swindon was taken (10 time points over a year, 4 of which were used for Illumina sequencing per hive (Illumina, Inc., San Diego, CA, USA)). Varroa samples were collected from the same three hives in the Swindon apiary alongside the honey bee samples.

### DWV detection assay

RNA extractions, RT-PCR and High Resolution Melt (HRM) analysis were carried out according to a previous study (Martin *et al.*, 2012). In brief, total RNA was extracted from whole worker honey bees using an RNeasy mini kit (Qiagen, Hilden, Germany), according to the manufacturer's instructions. RT-PCR via oligo dT priming using previously designed DWV primers (Highfield *et al.*, 2009) and subsequent HRM was carried out using Sensifast No-Rox One Step Kit (Bioline, London, UK). RNA was diluted to ~100 ng μl^−1^ and 1 μl of template RNA was added per reaction. The DWV load per worker honey bee was calculated according to the method developed by Highfield *et al.* (2009). The amount of RNA used for each RT-PCR reaction was normalised per bee and the DWV load per bee was calculated through a DWV cRNA standard curve conversion (y=−3.695x+32.744).

### Bioinformatics pipeline

**Figure 1:**
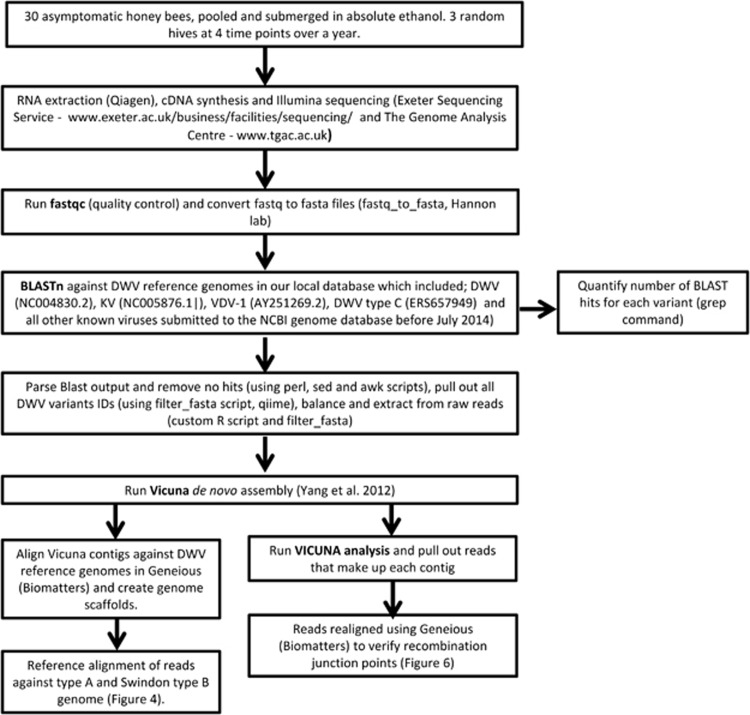
Bioinformatics pipeline leading to the application of the VICUNA *de novo* assembler.

Illumina Hi-seq (2 × 100 bp) pair-end sequencing was carried out by The Genome Analysis Centre (TGAC) and the University of Exeter. Total RNA was sequenced after a cDNA synthesis step with no amplification step necessary. Varroa RNA was pooled for three of the time points (January, April and May) prior to Illumina sequencing. A bioinformatics pipeline (Figure 1) was developed to accommodate the large amount of variation found within the DWV species complex. Firstly, the quality of the raw reads was verified using FastQC (Babraham Bioinformatics, Cambridge, UK). Samples were then converted from fastq to fasta using the fastq_to_fasta script which is part of the FASTX-toolkit (Hannonlab, http://hannonlab.cshl.edu/fastx_toolkit/). To isolate the reads sequenced from the DWV complex from the host and other contaminating sequences the BLASTn tool was used (Altschul *et al.*, 1990). The reads were searched against a custom BLAST database containing the DWV, VDV-1 and Kakugo virus genomes, with an e value of 10e-05. BLAST was carried out against Read 1 of the Illumina data. The ncbiblastparser perl script (http://www.bioinformatics-made-simple.com/2012/07/ncbi-blast-parser-extract-query-and.html) was then used to parse and read the top hit of the BLAST output. Next, ‘sed' and ‘awk' scripts were used to delete empty lines and the reads, which contained ‘nohits'. The corresponding BLAST hits were then pulled out from the Read 2 raw reads using QIIME. The paired reads were then balanced using a custom script written in R version 3.2.0 (R Core Team, 2015), which deletes any read in the Read 1 BLAST top hit file that did not have a pair in the corresponding read 2 BLAST top hit file and *vice versa*. The balanced DWV family reads were then assembled using the VICUNA assembler, which was developed to generate consensus assemblies from genetically heterogeneous populations, specifically RNA viruses (Yang *et al.*, 2012).

VICUNA contigs greater than 300 bp in length were imported into Geneious (Version 7.04, created by Biomatters) and the ‘Map to Reference tool' was used to align the contigs with the type A and B reference genomes. For several of the samples, the VICUNA assembly yielded full-length contigs that covered the whole genome, whereas others yielded only several smaller contigs (Supplementary Table S1). The ends of the contigs were then edited to remove discernable assembly or sequencing artefacts. Assembled DWV contigs were uploaded to the European Nucleotide Archive under the Study accession PRJEB8112. VICUNA contigs from hive 6 January 2013 were used to assemble a type B variant genome (accession number ERS754547).

The identity of the type B genome was compared with the VDV reference genome using the mVista tool (Supplementary Figure S1) (Frazer *et al.*, 2004), and the phylogeny of the Swindon variant was determined from a neighbour-joining tree of the polyprotein encoding region of the DWV genome (Lanzi *et al.*, 2006). In addition, genome scaffolding was carried out to produce full-length genomes representing the unique recombinant present in Swindon. SimPlot software was used to visualise the recombination event (Lole *et al.*, 1999).

To investigate the genome coverage of each DWV variant in Swindon, reads were grouped per hive (Varroa samples were all grouped together) and competitively aligned against the type A reference genome (NC004830.2) and the Swindon type B genome using the Geneious map to reference tool. The maximum percentage of mismatches per read accepted was 5% and no gaps per read were allowed.

To examine individual reads that make up the consensus sequence of each contig, the VICUNA analysis tool was used. To view the reads in Geneious, the VICUNA analysis output was modified by using a sed script to keep just the sequence reads. These were then converted from a tabular format into a fasta format using the python script ‘tab2fasta.py' and then visualised using Geneious. To quantify the number of reads with sequences similar to either DWV variant (type A or B), the Illumina reads were searched against a viral database using BLAST and the number of top hits attributed to each reference genome was quantified. Finally, genome coverage was calculated using the Lander/Waterman equation (read length × number of reads/genome length), which estimates the depth of sequencing across the genome (Sims *et al.*, 2014).

## Results and Discussion

Using a combination of RT-qPCR, HRM (Martin *et al.*, 2012) and Illumina (2 × 100 bp) Hi-seq sequencing (Figure 1), we investigated the DWV viral load and diversity in this small honey bee population in Swindon and their associated *Varroa* mites. Three hives were chosen at random and pools of 30 asymptomatic worker bees were sampled from inside the colony on 10 occasions at roughly monthly intervals between October 2012 and October 2013. RT-qPCR on an RNA-dependent RNA polymerase (*RdRp*) gene fragment for all 30 samples collected confirmed the persistence of high DWV loads (10^7^ to 10^8^ copies per bee) during the entire study period in all three hives (Supplementary Table S2). Both the DWV load and prevalence found within this study suggest that DWV presence alone cannot explain colony losses as proposed in previous Hawaiian (Martin *et al.*, 2012) and Devon, UK, studies (Highfield *et al.*, 2009).

**Figure 2:**
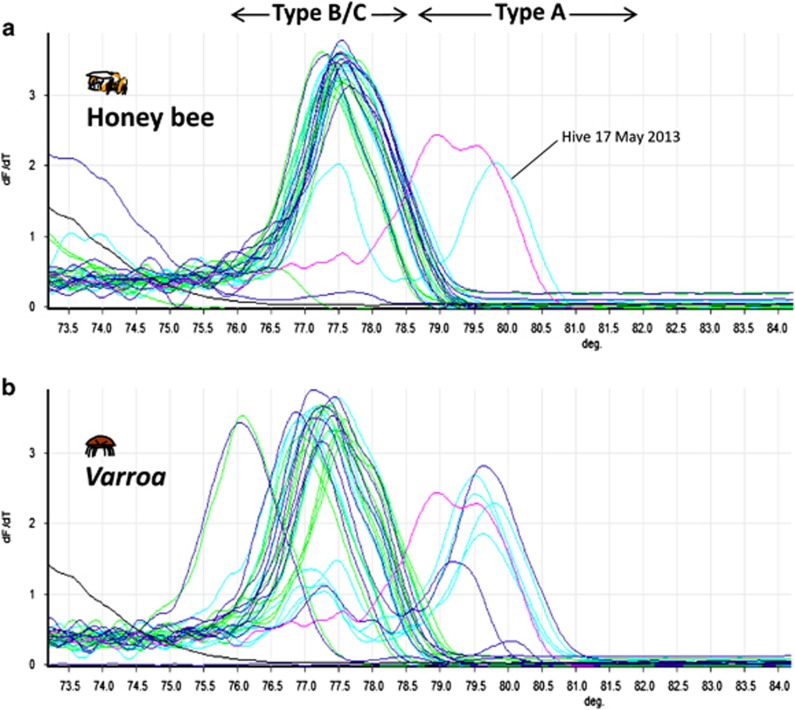
HRM curve analysis for DWV RdRp RT-qPCR region for three hives in the UK colonies (hive 6–blue, hive 17–cyan, hive 19–green). (**a**) Honey bees and (**b**) Varroa mites distinguishing between DWV type A and B/C variants. Deformed winged symptomatic bees were used as a positive control (pink line). A no template negative control was also run (black line).

To explore other factors that might contribute to this discovery, we exploited the known nucleotide polymorphisms in the *RdRp* gene fragment among the known DWV master variants (A, B and C) (Martin *et al.*, 2012; Mordecai *et al.*, 2015). HRM indicated the dominance of the type B or C master variant (Figure 2a), as these have similar melting temperatures (Martin *et al.*, 2012; Mordecai *et al.*, 2015). Only a single honey bee sample out of 30 tested contained both DWV type A and B/C, suggesting that although a colony can be exposed to type A, it fails to establish and neither persists nor accumulates. In contrast to the bee samples, the *Varroa* samples contained a greater mix of both DWV type A and B/C (Figure 2b), although type B/C remained the most prevalent. This prevalence of type B/C over A contrasts to what a previous study showed in Hawaii where the type A master variant dominated (Martin *et al.*, 2012) and suggests that type B/C may be an avirulent form of DWV. However, given that HRM analysis only detects limited genomic change (within the *RdRp* gene fragment), the possibility of recombination outside the RdRp region cannot be excluded. Both Mordecai *et al.* (2015) and Ryabov *et al.* (2014) showed that certain recombinants of the master variants A–C and A–B, respectively, could be more lethal than the type A master variant.

**Figure 3:**
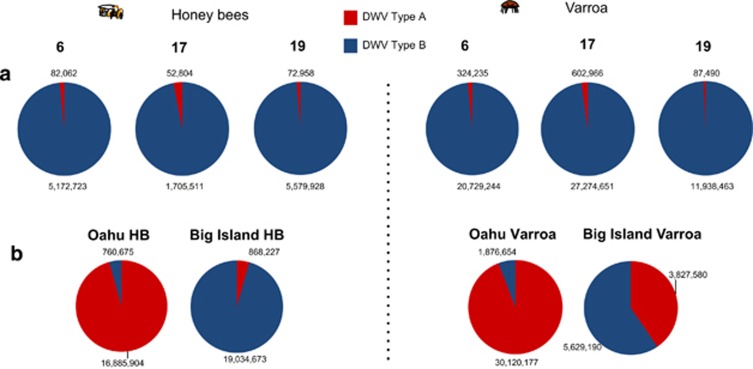
Proportions of DWV subgroups within colonies sequenced using Illumina Hi-seq. (**a**) Swindon samples collapsed into their respective hives 6, 17 and 19. (**b**) The Hawaiian samples from Oahu and Big Islands. A BLASTn algorithm against a custom DWV quasispecies database was used and the numbers indicate of hits to each DWV variant.

**Figure 4:**
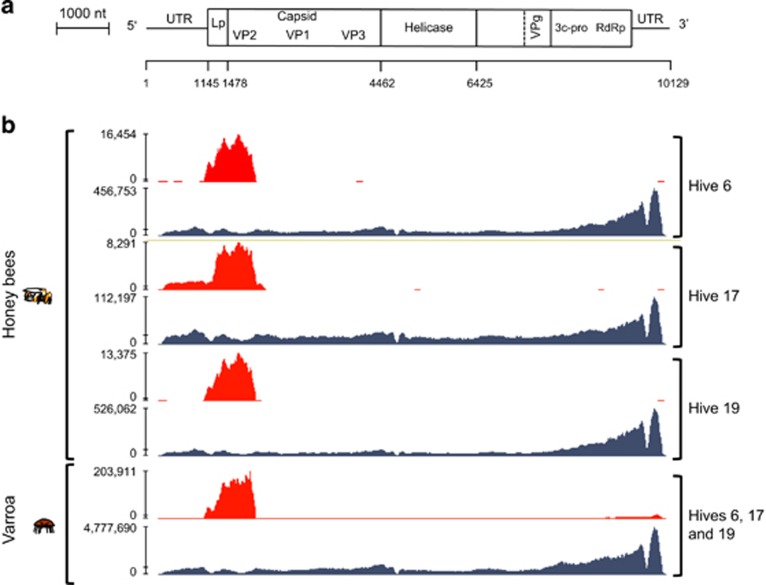
Genome coverage from the Illumina Hi-seq data for the Swindon colonies. (**a**) Map of the DWV genome adapted from Lanzi *et al* (2006). (**b**) DWV type A and B genomes (in red and blue, respectively) assembled from the Illumina NGS data from honey bees and mites from the Swindon apiary (hives 6, 17 and 19). *De novo* assembled VICUNA contigs that makeup these genomes for each hive were deposited in European Nucleotide Archive (ENA) under accession numbers ERS636096 to ERS636117.

**Figure 5:**
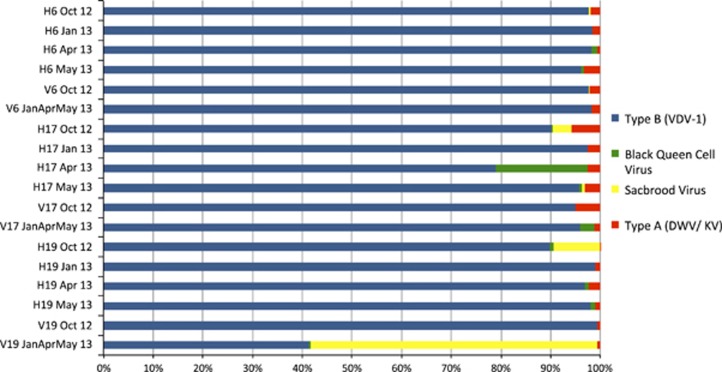
Virome of the Swindon apiary. Illumina reads were searched against a viral database (Figure 1) using BLASTn and the proportion of top hits associated with honey bee viruses was counted. DWV type B dominated the monthly samples in both the honey bee samples (H6, H17, H19) and the Varroa samples (V6, V17, V19).

DWV type B master variant dominance was, however, confirmed by Illumina sequencing (Figures 3,4,5). As a proportion of the total sequenced Illumina reads, DWV hits accounted for an average of 46.3% of reads in the *Varroa* samples and 9.7% of reads in the honey bees. The average DWV genome coverage for the honey bee samples was 22 484X, while the *Varroa* samples had an average DWV coverage of 599 558X. VICUNA assembly produced 6410 contigs across the 18 samples (Supplementary Table S1). Sample ‘Hive 6 January 2013' was used to assemble the ‘Swindon' DWV type B variant (Supplementary Table S3), which was found to be 99.5% identical to the type B reference genome (VDV-1) (Supplementary Figure S1). Figure 4 also shows that the type B DWV coverage was high, with over 15 million reads aligned from the honey bee samples compared with 241 000 reads aligned to the type A reference. Similarly, in the *Varroa* samples, 71.5 million reads aligned to type B compared with just over 1 million for type A. Type B reads aligned across the whole genome, whereas full genome coverage of type A was restricted to the *Varroa* samples. No reads unique to the Devon DWV type C genome could be found, whether in the honey bee or *Varroa* samples. In all, the honey bee- and *Varroa*-associated virome of the isolated UK study colonies was predominantly DWV type B (Figure 5), indicating that alternate DWV master variant competitive outcomes are possible.

**Figure 6:**
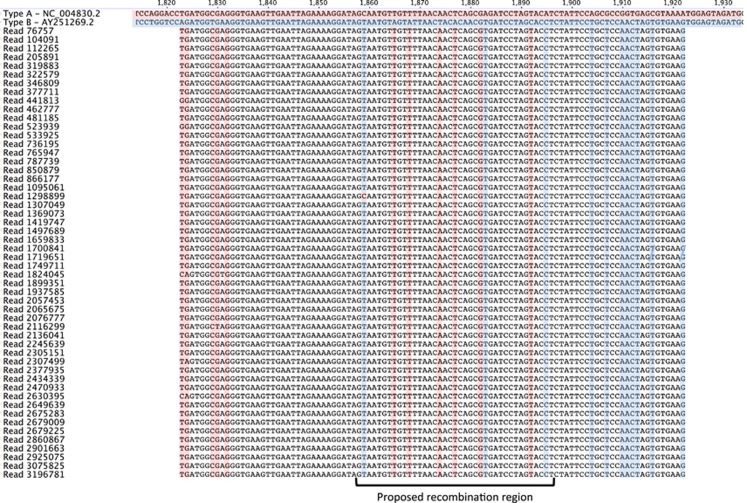
A multiple sequence alignment of the reads covering the recombination junction in the DWV recombinant from H19, April 2013. Output from VICUNA analysis was converted into a suitable format and imported into Geneious to visualise the reads over the type A–B recombination junction point. The DWV type A and B reference sequences are shown at the top and highlighted red and blue, respectively. Base pair substitutions common to either DWV type A and B variants are highlighted in each 100 bp Illumina read. In this example, 52 out of a total of 2464 reads is shown that covers the proposed recombination region.

**Figure 7:**
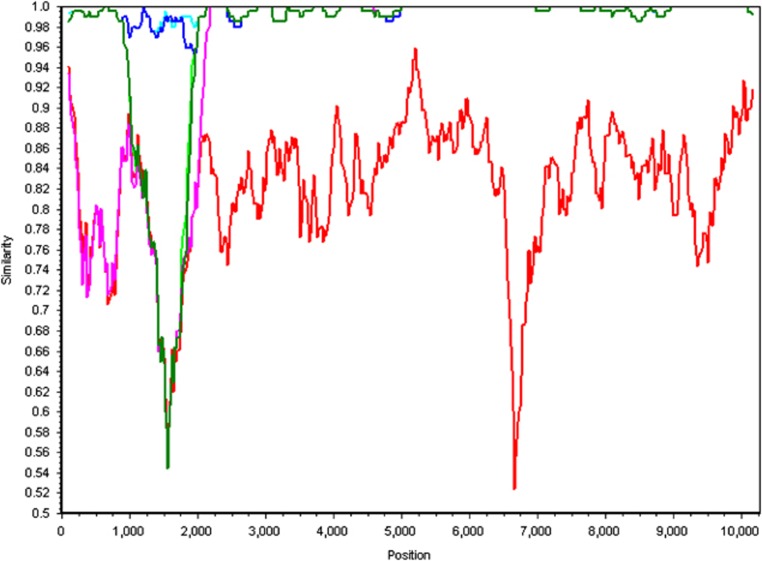
Simplot analyses of the different genomes present in the Swindon samples. Nucleotide similarities of various variants are compared with the type B (VDV) reference genome (AY251269.2). The type A (DWV) reference genome (NC004830.2) is shown in red. A selection of DWV genome scaffolds containing recombination in the 5′ end of the genome are shown; neon and dark green (type B-A-B recombinant from January 2013 Hives 17 and 6, respectively), cyan (Swindon type B genome from Hive 6 January 2013), dark blue (Swindon type B genome from Hive 17 January 2013) and pink (type A- B recombinant, H17 April 2013). A sliding window of 200 nt was used, moving in a step of 20 nt.

*De novo* and reference assembly of the DWV variant genomes suggested that recombination has taken place with type A possibly being recombined out, as evidenced by the presence of DWV recombinants within the honey bee samples (Figures 4, 6 and 7). Full genome scaffolds of each recombinant were made using the VICUNA contigs. These were aligned with type A and B genomes and Simplot (Figure 7) revealed that the recombination junction in the Swindon samples differed from that previously reported. Moore *et al.* (2011) showed that a recombination junction occurred in the 5′ untranslated region of the genome whereas the Swindon DWV type A-B recombinant junction found here occurs in the structural region of the open reading frame (Figures 4 and 7). Although full genome coverage was not achieved in both honey bee and *Varroa* samples by *de novo* assembly for type A, interestingly, reference alignment of DWV reads from the Swindon *Varroa* mites shows that the whole genome of type A is present at low levels (Figure 4), although HRM analysis indicated that the type A (master or any recombinants thereof) is rapidly removed in the following 5 months (see Figure 2, hive 17). A low number of type A reads (1.68% according to BLAST analysis) present in the UK study population (Figure 3) were uniquely associated with novel recombinants (Figures 4, 6 and 7, Supplementary Table S4) in which the majority of the genome were type B but contained a region of type A sequence at the 5′ end of the genome (the UTR and leader protein, Figure 4). The number of reads within the region of recombination for each of the hives was counted to compare the depth of coverage between the two variants (Supplementary Table S4). As this is a direct comparison of the same region of the genome, that is, the 3′ end, which is caused by bias in reverse transcription oligo dT priming (Figure 4), the 3′ bias is not relevant. In all hives, the number of type B reads exceeded the number of type A (recombinant) reads by an order of between 4.5 (Hive 19) and 36.4 (hive 17). Therefore, the dominance of type B master variant in this UK study population appears to be correlated with a level of colony protection as it appears to exclude type A or C (and any virulent recombinants thereof).

To compare this discovery of type B dominance in this study with respect to the previous Hawaiian study (Martin *et al.*, 2012), a small number of honey bee and *Varroa* Hawaiian samples with a known *Varroa* history were also subject to Illumina (2 × 100bp) Hi-seq sequencing. The same analytical VICUNA pipeline as that used for the UK samples (Figure 1) resulted in 212 contigs being assembled (Supplementary Table S1). On Oahu, where *Varroa* had established and caused widespread colony death, a colony analysed by Hi-seq (173 567X coverage) revealed that type A dominated (Figure 3b) confirming HRM data from another 28 colonies from Oahu, which also had predominantly type A (Martin *et al.*, 2012). However, in the colony from Big Island where *Varroa* had been present for less than 2 years and widespread colony collapse was yet to occur, type B dominated the sequence reads (195 760X coverage). In contrast, the *Varroa* sample from the same colony on Big Island contained a nearly equal mix of type A and B (93 014X coverage), whereas *Varroa* from Oahu (314 713X coverage) was dominated by type A (Figure 3b). A switch in dominance between type A and B in the Big Island honey bees suggests active competition between the two DWV variants consistent with the suggested 1–3-year time lag for DWV variants adapted to mite transmission to undergo selection (Martin *et al.*, 2012). As in Swindon, no significant matches to type C could be found in the honey bee or *Varroa* samples on either island. The time lag of the B to A switch in Big Island corresponds to the period when the mite becomes established but before colonies start dying. The normal outcome of this variant competition is the dominance of type A as evidenced by its transmission around the world (Berényi *et al.*, 2007). In the *Varroa*-resistant Swindon apiary, once established, the avirulent type B variant appears to prevent type A from becoming dominant. Crucially, in Swindon, the *Varroa* mites contained a proportion of type A reads (representing the whole type A genome) which were not detected in the honey bees suggesting that effective transmission of type A from parasite to host was prevented (Figure 4).

**Figure 8:**
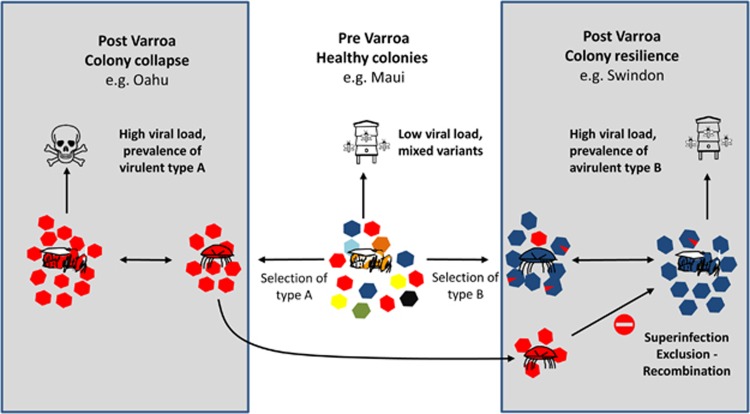
New honey bee-Varroa mite-DWV equilibrium. Type A DWV is represented in red and type B in blue. In Varroa-free hives, DWV exists as a cloud of variants present at low levels. In diseased hives such as Oahu, the type A is present in a Varroa-mediated transmission cycle. Whereas in Swindon, transmission of type B between bees and Varroa prevents the incursion of the type A variant into honey bees and consequently the hive survives.

SIE has been well documented in viruses related to DWV, for example, Tscherne *et al.* (2007) used cell lines to show that infection by one genotype of hepatitis C virus prevented infection by others. SIE best explains the phenomenon of why, despite high DWV load and *Varroa* infestation, the isolated UK colonies do not collapse. We speculate that co-evolution of the honey bee-*Varroa* mite-DWV system has selected for a new stable equilibrium where both the *Varroa* and an avirulent type B variant of DWV protect the honey bee, and thus the colony, from the virulent type A (Figure 8). Further work to validate this and determine the mechanism of the viral exclusion is required. For example, to demonstrate whether type B can protect against type A or C at the cellular and individual honey bee-level using assays similar to those described by Ryabov *et al.* (2014). If true, this would be the first report of SIE acting on the *Iflavirus* pathogens of bees. Ironically, it may be the presence of the mite population that is protecting the colony as *Varroa* may be providing the opportunity for constant re-introduction of type B into the population via horizontal transmission. In addition, although recombinants were present in both honey bee and *Varroa* samples, it is unclear whether these originate in the honey bees, *Varroa* or both.

It also remains unclear under what conditions type B can prevail or whether similar mechanisms of protection operate in the Brazilian, USA and French populations. Although the mechanism for exclusion seen in the Swindon apiary is unclear, a unique recombinant between type A and B was found (Figure 6) suggesting that the full-length type A genome (Figure 4) is actively suppressed. This is the counterpart of recombination causing acute infections as described by Moore *et al.* (2011) and Ryabov *et al.* (2014). Other candidate mechanisms have previously been identified in different viruses at various stages of the viral life cycle, including blocking of virus entry to the cell at the level of receptor interaction or occupation of sites for RNA replication (Lee *et al.*, 2005). Alternatively, the dominance of type B in the Swindon samples could be because of the induction of a differential immune response from the host such as RNAi (Hunter *et al.*, 2010).

Studies on honey bee pathogens have suggested that natural selection favours the survival and transmission of DWV over viruses of the Acute Bee Paralysis Complex (ABPV, KBV and IAPV), which have a higher virulence (de Miranda and Genersch, 2010; Schroeder and Martin, 2012). In this scenario, virus survival requires that the pupae live long enough to enable *Varroa* maturation and allow onward virus transmission. For example, the acute virulence of ABPV kills both adults and pupae quickly, ending the transmission cycle as mites associated with the pupae do not survive (Schroeder and Martin, 2012). The same reasoning can be applied to the DWV quasispecies where a particular host-variant dynamic dictates stable transmission or prevalence. Therefore, the Swindon UK population in question could have evolved to favour DWV type B persistence as a result of husbandry practices that have selected for a new stable non-pathogenic equilibrium. However, this phenomenon is not peculiar to Swindon as a recent study in South Africa found only DWV type B in four study apiaries, with no type A detected in either mites or honey bees (Strauss *et al.*, 2013). This raises the possibility that SIE may be operating on a wider scale in some geographical locations.

On the basis of our study, we hypothesise that within the swarm of DWV, owing to SIE, different viral variants are competing with two discernible outcomes. Either the disease-causing variants dominates, which can lead to colony collapse (Martin *et al.*, 2012), or an avirulent variant can prevail, reaching high viral loads which excludes the virulent variants. In the Swindon apiary, an evolutionary stable state has been reached in which disease symptoms are minimal and colonies survive. The data show that the dominance of type B in this isolated UK apiary has been stable only over a year of sampling, but anecdotal evidence suggests that the viral makeup of the bees at the Swindon Honey bee Conservation Trust has been stable for some time longer.

The discovery of a potential SIE mechanism in honey bees gives those wishing to limit or eradicate the sources of honey bee colony decline the possibility of active intervention. For example, in the citrus industry, where SIE is used to reduce crop losses by inoculating plants with a benign variant of *Citrus tristeza* virus to protect against infection by a more pathogenic form (Lee and Keremane, 2013). Accordingly, the direct introduction of DWV type B could provide a form of biocontrol against further collapse of European honey bee colonies in the face of *Varroa* infestation.
