# Supplementary material for: Superinfection exclusion and the long-term survival of honey bees in Varroa-infested colonies
Source: ISME J. 2015 Oct 27;10(5):1182–91. doi: 10.1038/ismej.2015.186 (PMC5029227; doi:10.1038/ismej.2015.186)
Supplement: Supplementary Table S1 [file ismej2015186x1.docx]

| **Sample name** | **Number of contigs** | **Maximum contig length** | **Average length of contigs** | **Number of reads used in all contigs** |
| --- | --- | --- | --- | --- |
| H6_Oct_12 | 230 | 4300 | 267 | 1,977,833 |
| H6_Jan_13 | 253 | 7757 | 261 | 1,556,582 |
| H6_Apr_13 | 241 | 5039 | 263 | 2,521,732 |
| H6_May_13 | 372 | 8734 | 249 | 2,347,253 |
| V6_Oct_12 | 405 | 3891 | 247 | 1,287,116 |
| V6_JanAprMay_13 | 764 | 4982 | 256 | 35,802,604 |
| H17_Oct_12 | 22 | 8622 | 711 | 136,123 |
| H17_Jan_13 | 53 | 5488 | 424 | 701,666 |
| H17_Apr_13 | 238 | 4274 | 267 | 1,905,055 |
| H17_May_13 | 18 | 3752 | 594 | 3289 |
| V17_Oct_12 | 654 | 5086 | 243 | 16,283,045 |
| V17_JanAprMay_13 | 902 | 10,449 | 251 | 30,620,382 |
| H19_Oct_12 | 52 | 3331 | 416 | 508,140 |
| H19_Jan_13 | 198 | 2642 | 270 | 1,314,217 |
| H19_Apr_13 | 388 | 10,253 | 247 | 3,671,238 |
| H19_May_13 | 362 | 5108 | 248 | 3,342,658 |
| V19_Oct_12 | 834 | 4374 | 253 | 14,793,194 |
| V19_JanAprMay_13 | 424 | 6179 | 248 | 4,579,032 |
| Oahu HB | ­41 | 6255 | 590 | 38,364,999 |
| Oahu Varroa | 69 | 6276 | 429 | 34,234,151 |
| Big Island HB | 62 | 10509 | 598 | 41,880,850 |
| Big Island Varroa | 40 | 12237 | 806 | 19,036,992 |

**Table S1** Contigs assembled from Hi-seq Illumina pair end reads for the Swindon samples
