# Supplementary material for: Superinfection exclusion and the long-term survival of honey bees in Varroa-infested colonies
Source: ISME J. 2015 Oct 27;10(5):1182–91. doi: 10.1038/ismej.2015.186 (PMC5029227; doi:10.1038/ismej.2015.186)
Supplement: Supplementary Table S2 [file ismej2015186x2.docx]

| **Table S2** DWV load per worker honey bee collected over the sampling period of October 2012 to October 2013 from the Swindon, UK, Apairy. | | | | | | | | | |  |
| --- | --- | --- | --- | --- | --- | --- | --- | --- | --- | --- |
| **Hive** | **Sampling Date** | **mg sample** | **RNA (ng/ul)** | **Elution vol** | **Dilution in qPCR** | **RNA per qPCR (ng)** | **Ct from qPCR** | **DWV Ct cDNA standard curve conversion** | **RNA (ng) per bee (bee = 100 mg)** | **DWV per bee** |
| H17 | Oct-12 | 50 | 42.4 | 30 | N/A | 42.4 | 20 | 3.45E+05 | 2544 | 2.07E+07 |
|  | Dec-12 | 50 | 31.5 | 30 | N/A | 31.5 | 17.45 | 4.14E+05 | 1890 | 2.48E+07 |
|  | Jan-13 | 50 | 152.7 | 30 | N/A | 152.7 | 19.05 | 3.71E+05 | 9162 | 2.22E+07 |
|  | Apr-13 | 50 | 120.8 | 30 | N/A | 120.8 | 17 | 4.26E+05 | 7248 | 2.56E+07 |
|  | May-13 | 50 | 443.3 | 30 | 1 in 4 | 110.8 | 16.56 | 4.38E+05 | 26598 | 1.05E+08 |
|  | Jun-13 | 50 | 941.3 | 30 | 1 in 9 | 104.6 | 16.58 | 4.37E+05 | 56478 | 2.36E+08 |
|  | Jul-13 | 50 | 413.8 | 30 | 1 in 4 | 103.45 | 19.25 | 3.65E+05 | 24828 | 8.76E+07 |
|  | Aug-13 | 50 | 93.5 | 30 | N/A | 93.5 | 24.36 | 2.27E+05 | 5610 | 1.36E+07 |
|  | Sep-13 | 50 | 238.9 | 30 | 1 in 2 | 119.45 | 18.19 | 3.94E+05 | 14334 | 4.73E+07 |
|  | Oct-13 | 50 | 274.9 | 30 | 1 in 2 | 137.45 | 16.48 | 4.40E+05 | 16494 | 5.28E+07 |
| H19 | Oct-12 | 50 | 390.9 | 30 | 1 in 3 | 130.3 | 18.7 | 3.80E+05 | 23454 | 6.84E+07 |
|  | Dec-12 | 50 | 142.4 | 30 | N/A | 142.4 | 16.37 | 4.43E+05 | 8544 | 2.66E+07 |
|  | Jan-13 | 50 | 92.9 | 30 | N/A | 92.9 | 16.18 | 4.48E+05 | 5574 | 2.69E+07 |
|  | Apr-13 | 50 | 51.8 | 30 | N/A | 51.8 | 17.15 | 4.22E+05 | 3108 | 2.53E+07 |
|  | May-13 | 50 | 119.1 | 30 | N/A | 119.1 | 17.08 | 4.24E+05 | 7146 | 2.54E+07 |
|  | Jun-13 | 50 | 476.5 | 30 | 1 in 4 | 119.1125 | 18.9 | 3.75E+05 | 28587 | 8.99E+07 |
|  | Jul-13 | 50 | 614.3 | 30 | 1 in 6 | 102.4 | 26.41 | 1.71E+05 | 36858 | 6.17E+07 |
|  | Aug-13 | 50 | 125.2 | 30 | N/A | 125.2 | 22.71 | 2.72E+05 | 7512 | 1.63E+07 |
|  | Sep-13 | 50 | 212.4 | 30 | 1 in 2 | 106.2 | 26.37 | 1.73E+05 | 12744 | 2.07E+07 |
|  | Oct-13 | 50 | 437.4 | 30 | 1 in 4 | 109.4 | 21.84 | 2.95E+05 | 6561 | 1.77E+07 |
| H6 | Dec-12 | 50 | 13.3 | 30 | N/A | 13.3 | 18.94 | 3.74E+05 | 798 | 2.24E+07 |
|  | Jan-13 | 50 | 108.2 | 30 | N/A | 108.2 | 19.38 | 3.62E+05 | 6492 | 2.17E+07 |
|  | Apr-13 | 50 | 311.8 | 30 | 1 in 3 | 103.9 | 18.97 | 3.73E+05 | 18708 | 6.71E+07 |
|  | May-13 | 50 | 543.6 | 30 | 1 in 5 | 108.7 | 18.41 | 3.88E+05 | 32616 | 1.16E+08 |
|  | Jun-13 | 50 | 87.0 | 30 | N/A | 87.0 | 17.68 | 4.08E+05 | 5220 | 2.45E+07 |
|  | Jul-13 | 50 | 1014.0 | 30 | 1 in 10 | 101.4 | 17.76 | 4.06E+05 | 60840 | 2.43E+08 |
|  | Aug-13 | 50 | 197.7 | 30 | N/A | 197.7 | 20.82 | 3.23E+05 | 11862 | 1.94E+07 |
|  | Sep-13 | 50 | 108.1 | 30 | N/A | 108.1 | 21.63 | 3.01E+05 | 6486 | 1.80E+07 |
|  | Oct-13 | 50 | 311.7 | 30 | 1 in 3 | 103.9 | 28.02 | 1.28E+05 | 18702 | 2.30E+07 |
