# Supplementary material for: Superinfection exclusion and the long-term survival of honey bees in Varroa-infested colonies
Source: ISME J. 2015 Oct 27;10(5):1182–91. doi: 10.1038/ismej.2015.186 (PMC5029227; doi:10.1038/ismej.2015.186)
Supplement: Supplementary Table S3 [file ismej2015186x3.docx]

| **Genome name** | **Number of contigs used to create genome scaffold** | **Average length of contigs** | **Total number of reads that make up genome** | **Percentage (%) of reads used in genome scaffold of total reads assembled by Vicuna** |
| --- | --- | --- | --- | --- |
| H6_Jan_13 Type B genome | 2 | 5245 | 1,310,540 | 84.2 |

**Table S3** Swindon DWV type B genome assembly
