# Supplementary material for: Superinfection exclusion and the long-term survival of honey bees in Varroa-infested colonies
Source: ISME J. 2015 Oct 27;10(5):1182–91. doi: 10.1038/ismej.2015.186 (PMC5029227; doi:10.1038/ismej.2015.186)
Supplement: Supplementary Table S4 [file ismej2015186x4.docx]

**Table S4** Sequence depth comparison for the recombination region**.** The start and end region represent the nucleotide where the recombinant type A sequence began and end for each hive on the DWV reference genome (gi|71480055) for type A variants and on the type B genome scaffold from Swindon, hive 6 (ERS754547).

| **Hive** | **Variant** | **Region Start** | **Region End** | **Number of reads** |
| --- | --- | --- | --- | --- |
| H6 | Type A | 762 | 1885 | 98,505 |
| H6 | Type B | 748 | 1858 | 470,062 |
| H17 | Type A | 0 | 2094 | 10,221 |
| H17 | Type B | 26 | 2067 | 372,801 |
| H19 | Type A | 852 | 1887 | 78,580 |
| H19 | Type B | 838 | 1860 | 361,345 |
