# Supplementary figures and images for: Superinfection exclusion and the long-term survival of honey bees in Varroa-infested colonies
Source: ISME J. 2015 Oct 27;10(5):1182–91. doi: 10.1038/ismej.2015.186 (PMC5029227; doi:10.1038/ismej.2015.186)

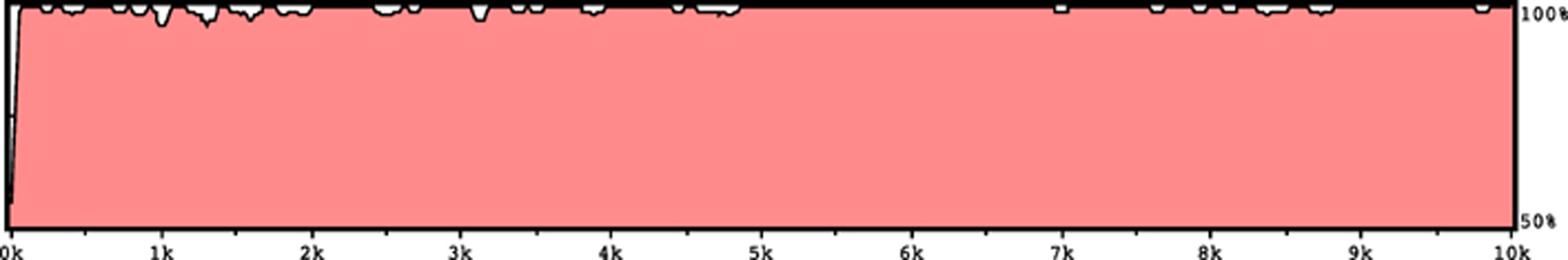

Supplement: Supplementary Figure S1 [file ismej2015186x5.tif]
