# Supplementary material for: Superinfection exclusion and the long-term survival of honey bees in Varroa-infested colonies
Source: ISME J. 2015 Oct 27;10(5):1182–91. doi: 10.1038/ismej.2015.186 (PMC5029227; doi:10.1038/ismej.2015.186)
Supplement: Supplementary Figure Legends [file ismej2015186x6.docx]

**Supplementary Figure**

**Figure S1.** Plot showing the percentage identity across the whole genome of the genome scaffold from Hive 6 January 2013 (ERS754547) compared to the type B VDV reference genome (AY251269.2). The two genomes are 99.5% identical.
